# Editorial: Frailty: Risks and management

**DOI:** 10.3389/fmed.2023.1100557

**Published:** 2023-01-17

**Authors:** Leonardo Bencivenga, Karolina Piotrowicz

**Affiliations:** ^1^Gérontopôle de Toulouse, Institut du Vieillissement, CHU de Toulouse, Toulouse, France; ^2^Department of Advanced Biomedical Sciences, University of Naples “Federico II”, Naples, Italy; ^3^Department of Internal Medicine and Gerontology, Faculty of Medicine, Jagiellonian University Medical College, Kraków, Poland

**Keywords:** frailty, older adults, geriatric syndrome, aging, geriatric medicine

The Research Topic “Frailty: Risks and management” aims to investigate the recent advances in the risk assessment and management of frailty in older adults, a growing major public health burden due to population aging, through relevant articles proposed by research groups from different countries.

The selected articles address the typical problems associated with this geriatric multifactorial condition from different perspectives and points of view, highlighting emerging epidemiological aspects alongside consolidated ones, analyzing risk factors specific to sex, reporting the potential role of new diagnostic tools and helping to propose the multidisciplinary approach as an essential resource in the care of elderly people.

Cognitive decline is extremely common among older adults, and cognitive frailty represents an emerging nosological entity that selectively associates it with frailty, as a potentially reversible syndrome (1). Focusing on a population of 1,390 older adults from the Geriatrics Service of the Centro Médico Naval in Peru, Vargas-Torres-Young et al. found that cognitive frailty and its specific components (cognitive impairment and modified Fried Phenotype criteria) were associated with higher risk of mortality, stimulating discussion on the role of interventions aimed at reversing this condition.

Interventions aimed at treating frailty are the subject of intense debate, given that their effectiveness is conditioned by multiple factors. At this regard, Coelho-Júnior and Uchida investigated the effects of resistance training programs on frailty status, physical performance, cognitive function and blood pressure in 60 Brazilian pre-frail and frail older adults, randomly allocated to low-speed and high-speed exercises. The results are particularly interesting because, although both resistance trainings reversed frailty status and enhanced physical performance, different patterns of improvement were observed between frailty degrees, with effects probably mediated by the heterogeneity of the aging process.

The use of diagnostic tools in the management of frailty is of great relevance, and muscle ultrasound is attracting increasing attention in the scientific community, especially in the assessment of sarcopenia (2). This tool is the topic of two manuscripts published in the present collection. Lv et al. enrolled 150 people aged ≥65 years from the First Hospital Affiliated to Nanjing Medical University who had undergone the anterior ultrasound of ulnar, vastus lateralis and anterior tibial muscles. The authors demonstrated that frailty phenotype (Fried's model) (3) was closely related to muscle thickness and quality, especially vastus lateralis muscle, and that muscle quality also deteriorated in the prefrailty stage, earlier than thickness. Bencivenga et al. employed Rockwood's Frailty Index (4) and ultrasound of rectus femoris plus vastus intermedius muscles of dominant arm to assess the association between frailty and muscle thickness in a population of 136 hospitalized older adults. The authors found that frailty index resulted significantly and independently associated with age and muscle thickness. Both studies stimulate discussion on the opportunity to consider muscle ultrasound as an additional imaging domain of frailty.

In recent years, several pieces of literature have been focusing on the variability patterns of measurable variables as measures of the altered state of homeostatic mechanisms underlying the development of frailty, especially in cardiovascular medicine (5). In this context, the review by Arantes et al. propose that heart rate variability can constitute a potential marker of frailty, as epiphenomenon of changes in cardiac autonomic modulation. They provide an overview on the tools to monitor the heart rate variability and summarize the evidence on its association with frailty.

In the era of personalized medicine, with a tailored preventive and therapeutic approach to older adults, studies on epidemiology that take into account the specific risk factors for frailty in individual countries and cultures are needed. Wang, Lv, et al., based on the results from 13,859 participants in the Chinese Longitudinal Healthy Longevity Study (CLHLS), reported a high prevalence of pre-frail and frail participants (54.1 and 26.3%, respectively) and provided a comprehensive insight into the epidemiology of this syndrome and related adverse outcomes. In their second article (Wang, Zhang, et al.) included in the present collection, a corresponding paper on the epidemiology of frailty, the authors presented a wide range of sex-specific contributors to frailty. Indeed, focusing on a group of 3,327 participants from the CLHLS, they reported risk factors that were common for both sexes and others more associated with the male or female sex. The protective effect of greater household income, higher level of physical activity and fresh fruit and vegetable consumption was shown for both sexes.

The two above-mentioned Chinese studies are accurately supplemented by the results of the prospective observational China Health and Retirement Longitudinal Study (CHARLS). Huang et al. found in their 2-year follow-up project that undertaking frequent intellectual activities (including playing Ma-jong, chess or cards, attending courses or surfing the web) corresponded with decreased risk of frailty syndrome in older adults aged 60 years and more. When considering frailty risk factors from a wider perspective, iatrogenic harms come to the fore. In their prospective cohort study of hospitalized patients treated with intravenous infusions, Cao et al. reported that the risk of frozen shoulder within 1 year of hospital discharge was as high as 5.2%. The risk factors for its onset included longer time of intravenous infusion, longer hospital stay, older age and comorbidities.

The current global economic and political crises recall the key role of socio-economic support on the state of health of the various age groups of the population, with a medium-long term impact. In this context the research question of the research proposed by Gao et al. on the relation between hunger in childhood and frailty in old age, seems particularly important. In their cross-sectional analysis of data obtained from the 2018 Chinese Longitudinal Healthy Longevity Survey, the authors showed that experience of childhood hunger was linked to frailty in late life, mediated by age and financial resources.

Taking advantage of the multidisciplinary approach, which is strongly advocated for the proper management of the complexity of frailty syndrome, geriatricians should go hand in hand with ophthalmologists when working with middle-aged and older patients. The opinion paper included in our collection by Crooke et al. presented a comprehensive scope of presbyopia as an opportunity for the timely detection of pre-frailty and frailty.

A key component of the aging process is represented by comorbidities, which, together with chronological age, constitute the main factors associated with frailty. Granata et al. propose a systematic review to evaluate the use of Clinical Frailty Scale, a screening tool based on clinical judgment (6), for frailty assessment, with a specific focus on chronic and noncommunicable diseases. From the 56 studies included, this tool was associated with a variety of disease-related characteristics, and was a good predictor of clinical outcomes, life expectancy, hospitalizations, and quality of life.

In summary, appreciating the high impact of frailty on national health systems, the articles in this Research Topics collection provide a meaningful and up-to-date scenario on some key aspects of this syndrome, also pointing out interesting potential innovations and stimulating new concepts. The Research Topic “Frailty: Risks and management” represents an important contribution to the body of scientific evidence in the field of geriatric medicine and also reveal current research gaps, stimulating ideas for future research on the topic.

## Author contributions

LB and KP wrote and approved the editorial. All authors contributed to the article and approved the submitted version.
